# Up- and down-modulation of liver cytochrome P450 activities and associated events in two murine malaria models

**DOI:** 10.1186/1475-2875-9-81

**Published:** 2010-03-22

**Authors:** Ana Cecilia AX De-Oliveira, Renato S Carvalho, Flavio HM Paixão, Hellen S Tavares, Luciana S Gueiros, Carolina M Siqueira, Francisco JR Paumgartten

**Affiliations:** 1 Laboratory of Environmental Toxicology, Department of Biological Sciences, National School of Public Health, Oswaldo Cruz Foundation, Rio de Janeiro, RJ, Brazil

## Abstract

**Background:**

The mechanisms by which malaria up and down-regulates CYP activities are not understood yet. It is also unclear whether CYP activities are modulated during non-lethal malaria infections. This study was undertaken to evaluate the time course of CYP alterations in lethal (*Plasmodium berghei *ANKA) and non-lethal (*Plasmodium chabaudi chabaudi*) murine malaria. Additionally, hypotheses on the association of CYP depression with enhanced nitric oxide (NO) production, and of CYP2a5 induction with endoplasmic reticulum dysfunction, enhanced haem metabolism and oxidative stress were examined as well.

**Methods:**

Female DBA-2 and C57BL/6 mice were infected with *P.berghei *ANKA or *P*. *chabaudi *and killed at different post-infection days. Infection was monitored by parasitaemia rates and clinical signs. NO levels were measured in the serum. Activities of CYP1a (ethoxyresorufin-*O*-deethylase), 2b (benzyloxyresorufin-*O*-debenzylase), 2a5 (coumarin-7-hydroxylase) and uridine-diphosphoglucuronyl-transferase (UGT) were determined in liver microsomes. Glutathione-S-transferase (GST) activity and concentrations of gluthatione (GSH) and thiobarbituric acid-reactive substances (TBARS) were determined in the liver. Levels of glucose-regulated protein 78 (GRP78) were evaluated by immunoblotting, while mRNAs of haemoxygenase-1 (HO-1) and inducible nitric oxide synthase (iNOS) were determined by quantitative RT-PCR.

**Results:**

*Plasmodium berghei *depressed CYP1a and 2b and induced 2a5 in DBA-2 mice. In *P.berghei*-infected C57BL/6 mice CYP activities remained unaltered. In both strains, GST and UGT were not affected by *P.berghei*. *Plasmodium c*. *chabaudi *depressed CYP1a and 2b and induced 2a5 activities on the day of peak parasitaemia or near this day. CYP2a5 induction was associated with over-expression of HO-1 and enhanced oxidative stress, but it was not associated with GRP78 induction, a marker of endoplasmic reticulum stress. *Plasmodium chabaudi *increased serum NO on days near the parasitaemia peak in both strains. Although not elevating serum NO, *P.berghei *enhanced iNOS mRNA expression in the liver.

**Conclusion:**

Down-regulation of CYP1a and 2b and induction of 2a5 occurred in lethal and non-lethal infections when parasitaemia rates were high. A contribution of NO for depression of CYP2b cannot be ruled out. Results were consistent with the view that CYP2a5 and HO-1 are concurrently up-regulated and suggested that CYP2a5 induction may occur in the absence of enhanced endoplasmic reticulum stress.

## Background

Several studies have shown that stimulation of host defense mechanisms against infections, as well as treatment with pro-inflammatory cytokines, modulate the expression and activity of cytochrome P450 enzymes (CYP), thereby modifying the kinetics of drugs and toxicants [1,2]. Along this line, it was reported that *Plasmodium berghei *infection depressed the total content of cytochrome P450s (CYPs) and the expression and activity of several CYP isoforms in the rodent liver [3-6]. Furthermore, it was recently shown that *P.berghei *ANKA malaria induced CYP2a5 activity [7]. Since the aforementioned studies evaluated CYP changes only at a nearly terminal stage of lethal malaria, it remains unclear whether up- and down-modulation of CYPs occur at earlier stages of lethal infections and in non-lethal infections as well.

The mechanism by which murine CYP2a5 and its human orthologous 2A6 are up- or down-modulated by infections and inflammatory stimuli remains largely obscure. Kirby and coworkers suggested that inducers of CYP2a5 have in common the property of causing oxidative injury to endoplasmic reticulum (ER), thereby producing an overexpression of GRP78 in hepatocytes [8,9]. Abu-Bakar *et al *[10], on the other side, suggested that CYP2a5 and 2A6 play a major role in the oxidative metabolism of bilirubin (BR), a breakdown product of haem. Since induction of HO-1 results in elevated levels of bilirubin, Abu-Bakar [10] advanced a hypothesis that a concurrent up-regulation of haem-oxygenase (HO) and CYP2a5 is critical for maintaining a balance between production and elimination of BR. In human *Plasmodium falciparum *and in rodent *Plasmodium berghei *malaria, intense haemolysis occurs and high levels of circulating haem may be present [11-13]. In human as well as in rodent cells, free haem excess up-regulates the expression of HO-1, the rate limiting enzyme in the process of converting potentially toxic free haem into equimolar amounts of carbon monoxide (CO), biliverdin and iron (Fe) [14,15].

It has been noted that nitric oxide (NO) causes a concentration-dependent inhibition of CYP activities in liver microsomes in vitro [16,17]. Based on these findings, and also on in vivo experiments in which iNOS inhibitors partially reversed LPS-induced decline of liver CYP activities, Khatsenko *et al *[16] advanced a hypothesis that NO overproduction mediates the decrease in CYP activities induced by immunostimulation. Nonetheless, the idea that NO mediates a general depression of CYP activities caused by inflammatory and infectious stimuli remains controversial. Sewer *et al *[18] reported that the down-modulation of murine CYP2c29, 2e1 and 3a11 by the *Escherichia coli *endotoxin in vivo occurs independently of NO production, a conclusion that is at variance with Khatsenko and coworkers' hypothesis. The same authors, however, observed a NO-dependent degradation of CYP2b proteins, a finding that reveals a possible mechanistic link between NO overproduction and inhibition of CYP2b during immunostimulation. In all aforementioned studies host defense responses were stimulated either by LPS or bacterial infections. So far no study has investigated whether depression of CYP1a and 2b activities correlates with periods of enhanced production of NO during the course of parasitic infections.

This study was undertaken to provide data on the time course of alterations of CYP2a5, 1a and 2b activities in the mouse liver during the evolution of lethal (*P.berghei *ANKA) and non-lethal (*Plasmodium chabaudi chabaudi*) malaria infections. Along this line, it was also examined whether down- modulation of CYP1a and 2b activities correlates with periods of NO overproduction during lethal and non-lethal infections. Additionally, the present study investigated whether the induction of CYP2a5 activity was associated with ER dysfunction (GRP78 protein overexpression), enhanced haem metabolism (HO-1 induction) and oxidative stress. The activity of uridine diphosphoglucuronyl transferase (UGT), an enzyme that plays a critical role in the elimination of bilirubin, was evaluated in *P.berghei*-infected mice as well [19].

## Methods

### Animals

Female DBA-2 and C57BL/6 mice, 7 to 10 weeks old, from the Oswaldo Cruz Foundation (FIOCRUZ) breeding stock were used. All mice were housed in standard plastic cages with stainless steel covers and white wood shavings as bedding. Temperature (23 ± 2°C), air relative humidity (approximately 70%) and photoperiod (lights on from 8:00 a.m. to 8:00 p.m.) were controlled in the animals' room. A commercial pellet diet (Nuvital CR1, Nuvilab^®^, Curitiba, PR, Brazil) and filtered tap water were provided ad libitum. The study protocol was approved by the Ethics Committee on the Use of Animals of the Oswaldo Cruz Foundation (CEUA-FIOCRUZ).

### Chemicals

Substrates benzyloxy- and ethoxy-resorufin, coumarin, p-nitrophenol, 1-chloro-2,4-dinitrobenzene, uridine diphospho glucuronic acid (UDPGA), GSH, thiobarbituric acid (TBA), butylated hydroxytoluene (BHT), dithiothreitol (DTT), EDTA, pyrazole, Bradford reagent, BSA, sulphanylamide, naphtylenediamine dichlorhydrate (NEED), nitrate reductase, sodium nitrate, β-NADPH, β-NADP, glucose-6-phosphate, glucose-6-phosphate dehydrogenase, and *E.coli *LPS (type 0127:B8) were from Sigma Chemical Co (St. Louis, MO, USA). All other chemicals used in the experiments were of high analytical grade.

### Parasites and infection

Parasites causing lethal (*P.berghei *ANKA strain) and non-lethal (*P.chabaudi chabaudi *AS strain) malaria infections in DBA-2 and C57BL/6 mice were used. Malaria-infected animals were inoculated by the i.p. route with 0.2 mL of PBS-diluted blood from a donor mouse containing either 10^6 ^(*P.berghei*) or 2 × 10^7 ^(*P.chabaudi*) parasitized erythrocytes (PE) per milliliter. The donor mice had been inoculated with a stabilate of the parasite kept at -80°C in Alsever's solution. For each malaria-infected mouse, an age-paired non-infected control animal of the same sex and strain was injected with 0.2 mL of PBS solution. After infection, mice were daily examined for clinical signs of illness and a small drop of blood was taken from the tail tip for determination of parasitaemia rates. The percentage of parasitized erythrocytes (% PE) was determined by examining thin blood smears stained by the Romanowski's method (Panótico Rápido, Laborclin^®^, Pinhais, PR, Brazil). Groups of infected mice and their matched non-infected controls were killed by cervical dislocation at different times after infection. After death, spleens and livers were removed as quickly as possible, freed from fat and extra tissue, weighed, frozen and kept at -80°C until further use.

### Preparation of subcellular fractions for phase I and II enzyme assays

#### Microsomal fraction I

Liver microsomal fraction I (MF I) was prepared as described previously [20], except for the use of 100 mM Tris 150 mM KCl buffer solution pH 7.4 instead of the sucrose solution. Aliquots of MF I were stored at -80°C until further use. MF I was used for the monooxygenase and UGT activities.

#### Microsomal fraction II

Liver microsomal fraction II (MF II) was used for determination of microsomal glutathione S-transferase (mGST) activity. It was prepared essentially as described for MF I, except for using 150 mM Tris pH 8.0 buffer solution to avoid cytosolic contamination [21]. Aliquots of MF II were stored at -80°C until further use.

#### Cytosolic fraction

Liver cytosolic fraction (CF) was prepared as recommended by Abel *et al *[22]. Briefly, liver was homogeneized in 10 mM Tris 250 mM sucrose 0.2 mM DTT 1 mM EDTA buffer solution pH 7.4 and centrifuged at 10 000 × *g *for 10 min at 4°C. The supernatant was then centrifuged at 15 000 × *g *for 20 min at 4°C, filtered in gauze and centrifuged at 105 000 × *g *for 1 h at 4°C. Aliquots of CF were stored at -80°C until further assay of the cytosolic GST (cGST) activity.

### Total protein determination

Protein concentration in MF I, MF II and in CF was determined using Bradford reagent and BSA as the standard [23]. The method was adapted to a microplate and absorbance was read at 595 nm in a spectrophotometer Spectramax Plus^® ^(Molecular Devices, USA).

### Enzyme assays

#### Monooxygenase reactions

Benzyloxy-(BROD) and ethoxy- (EROD) resorufin-*O*-dealkylases were determined in 96-well microplates as described by Kennedy and Jones [24], with some modifications [7]. Substrate final concentration was 5 μM and 0.025 mg of microsomal protein was added to each well (MF I). After a 10-min incubation period at 37°C, 100 μL of acetonitrile was added to each well and the amount of resorufin was measured in a fluorescence plate reader (*Spectramax Gemini XS*^®^, Molecular Devices, USA) with excitation and emission wavelengths set at 530 and 590 nm, respectively.

#### Coumarin hydroxylase (COH) activity

COH activity was assayed as reported by Iersel *et al *[25] with a few adaptations. 50 mM Tris buffer pH 7.4, 10 μM coumarin and 0.4 mg (DBA-2) or 0.8 mg (C57BL/6) of protein was added to reaction tubes for a final volume of 0.5 mL. After a 3 min pre-incubation period, reaction was initiated by addition of a NADPH regenerating system (0.5 mM β-NADP, 10 mM glucose 6-phosphate, 0.5 U/mL glucose 6-phosphate dehydrogenase and 10 mM magnesium chloride). The reaction took place for 10 min at 37°C with shaking and was stopped by addition of HCl 2N. The reaction product (umbelliferone or 7-hydroxycoumarin) was extracted with chloroform and taken to tubes containing 1.6 M glycine-NaOH solution pH 10.4. The amount of umbelliferone was measured by using a spectrofluorimeter Shimadzu RF5301 PC^® ^with excitation and emission wavelengths set at 355 nm and 460 nm, respectively, and a 3 nm band slit width.

### Conjugation reactions

#### UGT activity

UGT activity was determined as reported by Bock *et al *[26]. 100 mM Tris pH 7.4, 0.25% triton X-100, 5 mM p-nitrophenol, 50 mM MgCl_2 _and 0.1 mg of protein (MF I) were added to reaction tubes to a final volume of 0.5 mL. After a 2 min pre-incubation period, UDPGA (3 mM) was added to start the reaction. After 30 min, trichloroacetic acid 5% was added to stop the reaction and the samples were centrifuged at 2 300 rpm in a bench centrifuge. 1 mL of supernatant was added to a cuvette that received 250 μL of 2 N NaOH. Samples were carried to a spectrophotometer Shimadzu UV 1601^® ^set at 405 nm and the enzyme activity was calculated using the molar extinction coefficient of 14.9 mM^-1 ^cm^-1 ^settled by Martin and Black [27].

#### GST activities

GST activities in microsomal (MF II) and cytosolic (CF) fractions were determined as described in details by Habig *et al *[28]. Briefly, 100 mM K_2_HPO_4 _buffer solution pH 6.5, 1 mM 1-chloro-2,4-dinitrobenzene, 1 mM glutathione, 5 μg (CF) or 50 μg (MF II) of protein and 0.1% Triton X-100 (MF II only) were added to the cuvette (final volume of 1 mL) where the reaction took place. The differential absorbance was recorded using a spectrophotometer Shimadzu UV 1601^® ^(set at 340 nm) and the amount of conjugate formed was calculated using the molar extinction coefficient of 9.6 mM^-1 ^cm^-1 ^[28].

### Measurement of GSH levels

After mouse death, the liver was removed as quickly as possible, washed in iced PBS, dried and homogenized in 100 mM NaPO_4 _5 mM EDTA buffer solution [29]. The hepatic homogenate was centrifuged at 10 000 × *g *at 4°C for 15 min and the supernatant was frozen and kept at -70°C until further use. After thawing, the supernatant (10 μL) was incubated with 12.5 μL of 25% phosphoric acid and 37 μL of 0.1 mM NaPO_4 _5 mM EDTA buffer solution pH 8.0 for 10 min at 4°C. It was centrifuged again at 13 000 × *g *for 10 min at 4°C, and the newly obtained supernatant was incubated with 0.1% o-phthaldialdehyde methanol solution and the same basic buffer solution for additional 15 min at room temperature. GSH concentration was then measured using a spectrofluorimeter (Shimadzu RF 5301 PC^®^) with wavelength parameters set at 350 nm (excitation), 420 nm (emission) and 3 nm (band slit width) and GSH standard curve [30,31].

### Thiobarbituric acid reactive substances (TBARS) assay

TBARS were measured as described by Hermes-Lima *et al *[32]. Livers were homogenized in a 0.2% phosphoric acid solution (1:5, w/v) and in another volume of a 2.0% phosphoric acid solution. 0.4 mL aliquots of homogenized tissue received the same volume of either 10 μM butylated hydroxytoluene (BHT) 1% thiobarbituric acid (TBA) - samples - or 3 mM HCl - blanks (each sample having its own blank). Both tubes received 0.2 mL of 7% phosphoric acid and were heated in boiling water for 15 min. After cooling at room temperature, the tubes received 1.5 mL of butanol, were vortex vigorously for 40 sec and centrifuged for 10 min. Two distinct phases appeared, an organic phase on the top (TBA-MDA complex) and an aqueous phase on the bottom. The absorbance of organic phase supernatant was read at 532 nm and 600 nm while blanks were scanned between 400 and 600 nm. To calculate malondialdehyde (MDA) concentration, absorbance at 600 nm was subtracted from that at 532 nm and a molar absorption coefficient of 156 mM^-1^cm^-1 ^was used [32].

### Nitric oxide (NO) levels in blood serum

NO production was evaluated by measuring total nitrite in serum as described in details elsewhere [33]. Briefly, nitrate was reduced to nitrite by incubating the serum sample with a nitrate reductase and the total amount of nitrite was then determined by the Griess method. Results are expressed as μM concentration of NO_2_^-^.

### Reverse transcriptase-polymerase chain reaction (RT-PCR) analysis (iNOS, HO-1)

Levels of mRNA in the liver and spleen were determined by real time RT-PCR as follows. RNA was extracted using Trizol^® ^reagent, quantified in a Nanodrop^® ^spectrophotometer, used (3 μg) as template to cDNA synthesis. The cDNA synthesis reaction was conducted in a Mastercycler Gradient thermocycler (Eppendorff^®^) using Superscript II^® ^system (Invitrogen^®^) and oligo DT primers (Promega^®^). Relative expressions of iNOS and HO-1 were determined using Taqman^® ^assays (Applied Biosystems^®^). Assays Mm00440485_m1 and Mm00516007_m1 were used for iNOS and HO-1 respectively, and the assay 4352341E was used for β-actin (endogenous control). Real time reactions were conducted in a 7500 Fast real time thermocycler (Applied Biosystems^®^). The relative quantification of the target genes was made using the ΔΔCt method.

### Gel electrophoresis (SDS-PAGE) and immunoblotting analysis (GRP78 protein)

SDS-PAGE was performed using 8% polyacrilamide resolving gel as described by Laemmli [34]. Microsomal proteins were separated by applying a constant current (30 mA) onto the gel (Hoefer miniVE system, GE Healthcare^®^). After separation, microsomal proteins were transferred into nitrocellulose membranes (Hybond ECL, GE Healthcare^®^) using the Hoefer miniVE semi-dry system (GE Healthcare^®^). Primary antibody was a rabbit polyclonal IgG (SC 13968, Santa Cruz Biotechnologies^®^, lot # F2206) against an epitope corresponding to amino acids 525-653 mapping at the C-terminus of the 78 kDa glucose-regulated protein (GRP78) of human origin. The blots were developed using a commercially available alkaline phosphatase kit (BIORAD^® ^AP conjugate substrate kit).

## Results

### Modulation of liver CYP activities during *P. berghei *(lethal) infection

**Figure 1:**
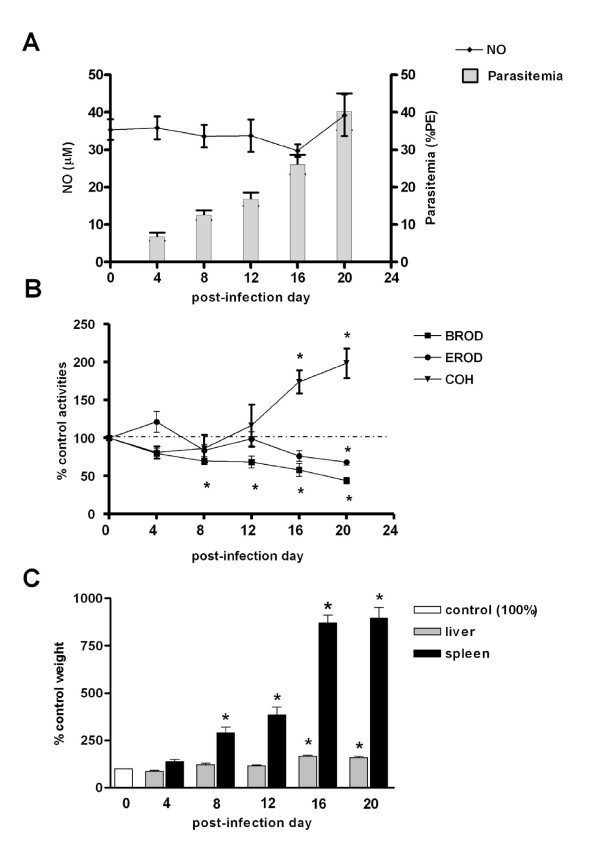
**Effects of *P.berghei *infection on CYP activities, NO production and liver weights in DBA-2 mice**. Time course of changes of liver CYP activities (CYP1a: EROD, 2b: BROD, 2a5: COH), NO serum levels, and liver and spleen weights in DBA-2 mice infected with *P.berghei *(ANKA). Panel A shows NO serum levels (μM) and parasitaemia rates (%PE). Results in Panel B (enzyme activities) and C (liver and spleen weights) are shown as % of mean values for non-infected controls (day 0 = 100) and comparisons were made by ANOVA and Dunnett's post-hoc test. In panels B and C statistical evaluation was made with untransformed data. Differences (P < 0.05) from non-infected control group (day 0) are indicated by an asterisk (*) superscript. Panels B and C control values (100%) are as follows (mean ± S.E.M.): EROD = 94.7 ± 41 pmol resorufin/mg protein/min; BROD = 74.6 ± 21 pmol resorufin/mg protein/min; COH = 193.9 ± 27 pmol umbelliferone/mg protein/min; liver wt = 0.99 ± 0.02 g; spleen wt = 0.08 ± 0.04 g. Control NO = 35.4 ± 2.8 μM (Panel A). Numbers of mice used (N) are as follows: PID0 = 15; PID4 = 10; PID8 = 10; PID12 = 10; PID16 = 11; PID20 = 14. Of 9 infected mice that were not killed (not included), 2 died on PID22, and 7 on PID24. No infected DBA-2 exhibited neurological signs of cerebral malaria.

**Figure 2:**
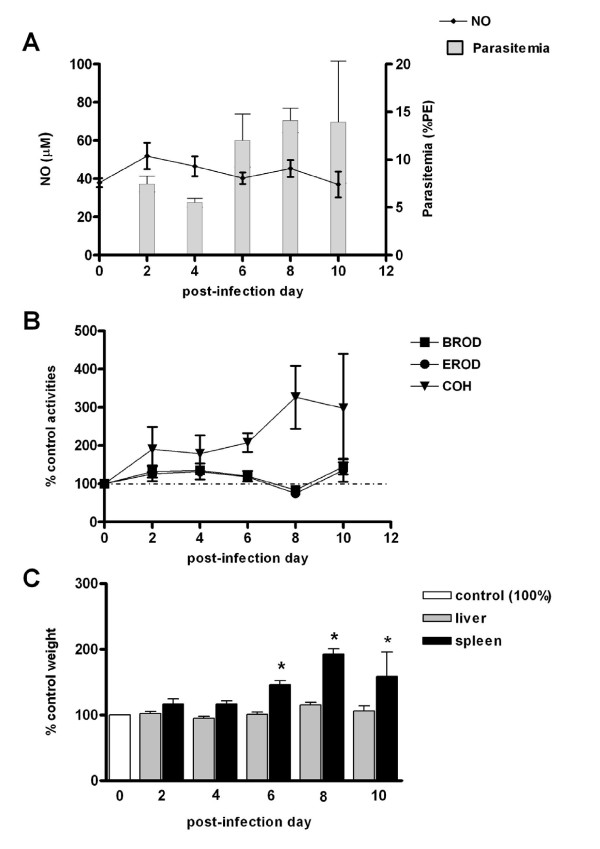
**Effects of *P.berghei *infection on CYP activities, NO production and liver weights in C57BL/6 mice**. Time course of changes of liver CYP activities (CYP1a: EROD, 2b: BROD, 2a5: COH), NO serum levels, and liver and spleen weights in C57BL/6 mice infected with *P.berghei *(ANKA). Panel A shows NO serum levels (μM) and parasitaemia rates (%PE). Results in Panel B (enzyme activities) and C (liver and spleen weights) are shown as % of mean values for non-infected controls (day 0 = 100) and comparisons were made by ANOVA and Dunnett's post-hoc test. In panels B and C statistical evaluation was made with untransformed data. Differences (P < 0.05) from non-infected control group (day 0) are indicated by an asterisk (*) superscript. Panels B and C control values (100%) are as follows (mean ± S.E.M.): EROD = 103.1 ± 33.5 pmol resorufin/mg protein/min; BROD = 87.3 ± 12.3 pmol resorufin/mg protein/min; COH = 9.81 ± 2.5 pmol umbelliferone/mg protein/min; liver wt = 0.99 ± 0.05 g; spleen wt = 0.09 ± 0.01 g. Control NO = 37.95 ± 2.4 μM (Panel A). Numbers of mice used (N) are as follows: PID0 = 5; PID2 = 7; PID4 = 7; PID6 = 7; PID8 = 7; PID10 = 3. Numbers of infected mice which died (not included): PIDs8/9 = 5, PIDs9/10 = 13. From day 8 onwards, all infected mice that were killed or died presented neurological signs of CM.

*Plasmodium berghei *caused a severe and deadly illness in DBA-2 and C57BL/6 mice. In DBA-2 mice, parasitaemia rose steadily from post-infection day (PID) 4 onwards, reaching rates as high as 40% PE on PID20 (Figure 1A). Clinical signs such as piloerection, tachypnea and hypoactivity became progressively more frequent and severe as parasitaemia rate increased. From PID8 onwards, spleens of infected mice were darker and markedly enlarged, being almost 10-fold heavier than non-infected mice's spleens on PID20. Livers from infected DBA-2 mice were also darker and somewhat larger than those from non-infected controls on PIDs 16 and 20 (Figure 1C). The clinical course of infection in C57BL/6 mice differed from that noted in the DBA-2 strain (Figure 2). Around PID10, when parasitaemia rates were nearly 15-20% PE (Figure 2A), most infected C57BL/6 mice exhibited neurological signs (ataxia, rolling and other signs of motor impairment) consistent with cerebral malaria which rapidly progressed to death. On PIDs 6, 8 and 10 C57BL/6 mice's spleens were enlarged (1.5 to 2-fold heavier than controls' spleens) and darker but no hepatomegaly was detected (Figure 2C). A remarkably high susceptibility of *P.berghei-*infected C57BL/6 mice to develop cerebral malaria was noted in other studies as well [35].

Alterations of liver monooxygenase activities during *P.berghei *infection are also shown in Figures 1 (DBA-2) and 2 (C57BL/6). The effects of *P.berghei *infection on DBA-2 liver enzymes were not examined beyond PID20 because mortality was high after this day. As shown in Figure 1, activities of CYP2a5 and 1a were up and down-regulated, respectively, only at the terminal stage of infection (EROD: PID20; COH: PID16 and 20). The activity of CYP2b, however, showed a progressive depression - from PID8 on - as infection evolved to a more severe stage (Figure 1B).

Since *P.berghei *infection in C57BL/6 mice evolved rapidly to death, it was not possible to evaluate eventual changes of CYP-mediated activities at higher parasitaemia rates. On PIDs 8 and 10, CYP1a and 2b activities remained nearly unchanged after infection while a non-significant increase in CYP2a5 activity was noted (Figure 2B).

### Changes of CYP activities in *P. chabaudi*- infected mice (non-lethal malaria)

**Figure 3:**
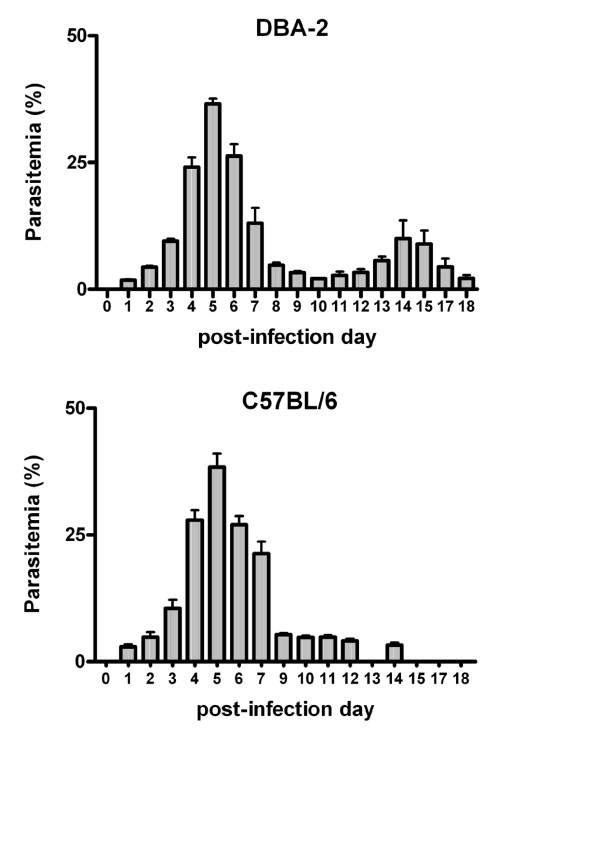
**Time course of non-lethal malaria (*P.chabaudi chabaudi*) infection in female DBA-2 and C57BL/6 mice**. Parasitaemia rates (%PE) were determined following inoculation of *P*. *c*. *chabaudi *(2 × 10^7 ^PE i.p.) on infection day 0 (PID0). Bar heights are means ± S.E.M. of % of parasitized erythrocytes (%PE). Number of mice used (N): DBA-2 = 5; C57BL/6 = 5.

**Figure 4:**
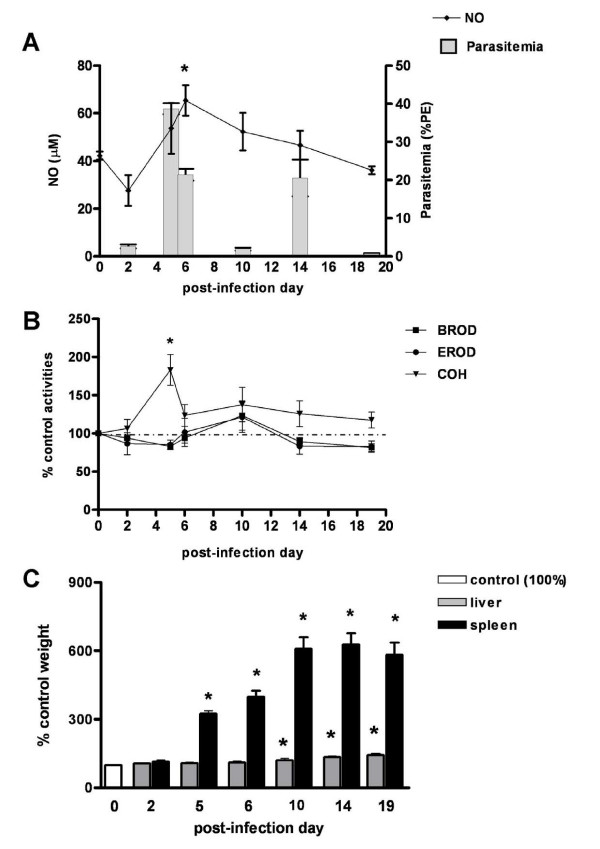
**Effects of *P. c. chabaudi *infection on CYP activities, NO production and liver weights in DBA-2 mice**. Time course of changes of liver CYP activities (CYP1a: EROD, 2b: BROD, 2a5: COH), NO serum levels, and liver and spleen weights in DBA-2 mice infected with *P.chabaudi chabaudi*. Panel A shows NO serum levels (μM) and parasitaemia rates (%PE). Results in panels B (enzyme activities) and C (liver and spleen weights) are shown as % of mean values for non-infected controls (day 0 = 100) and comparisons were made by ANOVA and Dunnett's post-hoc test. In panels B and C statistical evaluation was made with untransformed data. Differences (P < 0.05) from non-infected control group (day 0) are indicated by an asterisk (*) superscript. Panels B and C control values (100%) are as follows (mean ± S.E.M.): EROD = 130.8 ± 6.7 pmol resorufin/mg protein/min; BROD = 73.7 ± 3.1 pmol resorufin/mg protein/min; COH = 149.4 ± 8.0 pmol umbelliferone/mg protein/min; liver wt = 1.08 ± 0.01 g; spleen wt = 0.11 ± 0.005 g. Control NO = 42.25 ± 1.7 μM (Panel A). Numbers of mice used (N) are as follows: PID0 = 8; PID2 = 8; PID5 = 8; PID6 = 8; PID10 = 8, PID14 = 8; PID19 = 8.

**Figure 5:**
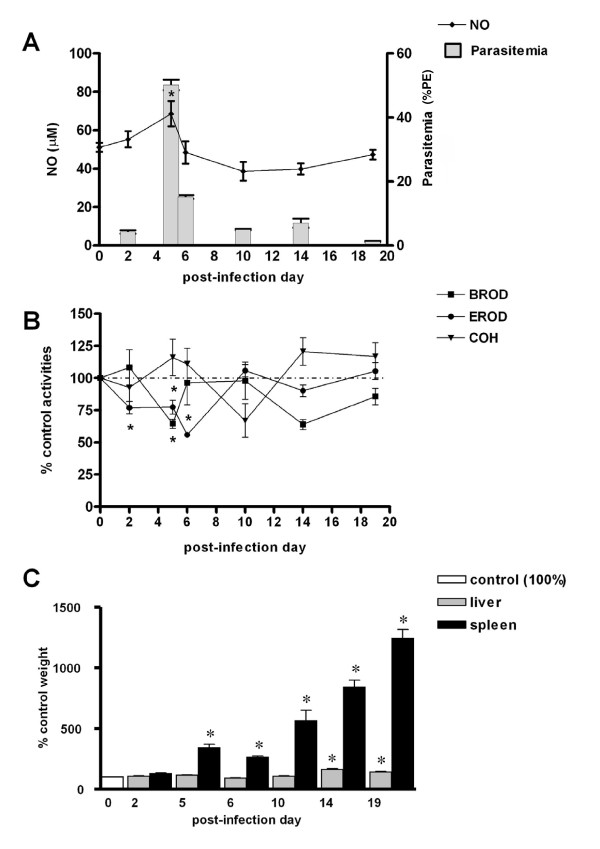
**Effects of *P. c. chabaudi *infection on CYP activities, NO production and liver weights in C57BL/6 mice**. Time course of changes of liver CYP activities (CYP1a: EROD, 2b: BROD, 2a5: COH), NO serum levels, and liver and spleen weights in C57BL/6 mice infected with *P*. *chabaudi chabaudi*. Panel A shows NO serum levels (μM) and parasitaemia rates (%PE). Results in Panel B (enzyme activities) and C (liver and spleen weights) are shown as % of mean values for non-infected controls (day 0 = 100) and comparisons were made by ANOVA and Dunnett's post-hoc test. In panels B and C statistical evaluation was made with untransformed data. Differences (P < 0.05) from non-infected control group (day 0) are indicated by an asterisk (*) superscript. Panels B and C control values (100%) are as follows (mean ± S.E.M.): EROD = 182.4 ± 5.8 pmol resorufin/mg protein/min; BROD = 121.2 ± 6.0 pmol resorufin/mg protein/min; COH = 16.5 ± 1.2 pmol umbelliferone/mg protein/min; liver wt = 0.84 ± 0.02 g; spleen wt = 0.07 ± 0.003 g. Control NO = 51.12 ± 2.36 μM (Panel A). Numbers of mice used (N) are as follows: PID0 = 10; PID2 = 14; PID5 = 10; PID6 = 6, PID10 = 6; PID14 = 6; PID19 = 8.

The time course of *P*. *chabaudi *infection in DBA-2 and C57BL/6 mice was evaluated in a preliminary experiment. In both strains, parasitaemia rates gradually increased after infection, reached a maximum rate (approx. 40% PE) on PID5 and declined to 2-5% PE on PID10 (Figure 3). A secondary peak of parasitaemia (10% PE) on PID14 appeared only in DBA-2 mice. No deaths were noted among *P*. *chabaudi*-infected mice and, except for piloerection on PIDs 5-6 (parasitaemia peak), infection evolved without major signs of illness. In both strains, from the day of parasitaemia peak (PID5) onwards spleens became markedly enlarged. Livers of infected DBA-2 and C57BL/6 mice were also darker and enlarged on PIDs 10-19 and 14-19, respectively (Figures 4A, 4C, 5A and 5C).

In the DBA-2 strain of mice, an induction of CYP2a5 activity (COH) was found on the day of peak parasitaemia (PID5) only, while activities of CYP1a (EROD) and 2b (BROD) remained unaltered at all times after infection (Figure 4B). In C57BL/6 mice infected with *P.chabaudi*, however, the activity of CYP2a5 (COH) was not altered during the whole course of the infection, although the activities of CYP1a (EROD) and 2b (BROD) were depressed around the day of peak parasitaemia, PIDs 2-6 and PID5, respectively (Figure 5B).

#### Nitric oxide (NO) production during the course of *P. berghei *and *P. chabaudi *infections

**Figure 6:**
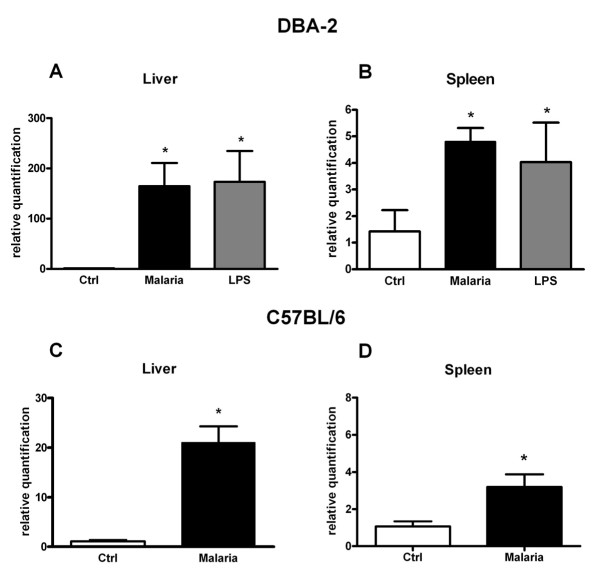
**Effects of *P. berghei *infection on iNOS mRNA expression in mice liver and spleen**. Expression of iNOS mRNA in the liver and spleen of DBA-2 (A and B, N = 3) and C57BL/6 (C and D, N = 4) mice infected with *P*. *berghei *(ANKA), when parasitaemia rates rose to levels >30%PE (DBA-2) on PID20 or >20%PE (C57BL/6) on PID10 and respective non-infected controls (N = 3). For comparative purposes, levels of iNOS mRNA were also determined in the liver (A, N = 6) and spleen (B, N = 5) of non-infected DBA-2 mice treated with *E*. *coli *LPS (5 mg/kg i.p. 12 hours earlier). Column height represents mean ± S.E.M. for relative levels of mRNA. Differences (Mann-Whitney U test, P < 0.05) between infected (malaria) and non-infected control (Ctrl) are indicated by an asterisk (*). Quantification of mRNA was by real time RT-PCR.

A slight (<two-fold) increase in serum levels of NO was observed on the day of *P.chabaudi *parasitaemia peak (PID5) in C57BL/6 (Figure 5A), or after this day (PID6) in DBA-2 mice (Figure 4A). During the course of *P.berghei *infection no increase in serum levels of NO was observed in either mouse strain used in this study (Figures 1A and 2A). Results, therefore, indicated that, as far as DBA-2 mice infected with *P.berghei *are concerned, depression of CYP1a and 2b activities in the liver occurred in the absence of any discernible increase in NO serum levels. Nonetheless, results showed that expression of iNOS mRNA in livers and spleens was enhanced in DBA-2 and C57BL/6 mice infected with *P*. *berghei *(Figure 6). As shown in Figure 6A, up-regulation of liver iNOS by *P.berghei *infection was comparable to that observed in DBA-2 mice that had been treated 12 hours earlier with LPS (5 mg/kg body wt i.p.).

#### Activity of GST and UGT, and levels of GSH and TBARS in the liver of *P. berghei-infected *mice

Activities of cytosolic and microsomal GSTs as well as the activity of UGT remained nearly unaltered in the liver of *P.berghei*-infected DBA-2 and C57BL/6 mice (Table 1). Results also showed that *P.berghei *infection produced a marked reduction of hepatic levels of GSH in C57BL/6 but not in DBA-2 mice. Levels of substances reacting with thiobarbituric acid (TBARS), however, were increased in DBA-2, but not in C57BL/6 mice (Table 1), a finding that seems to indicate that infection enhanced liver lipids peroxidation and oxidative stress in the former but not in the latter strain.

**Table 1:** Phase-II enzyme activity, glutathione and TBARS in the liver of *P.berghei*-infected mice

Parameters		GST		UGT	GSH	TBARS
**Sub-cellular fraction**		**m**	**c**	**m**	**c**	**-**

DBA-2	Infected (N)	80.4 ± 4.5 (8)	700 ± 100 (8)	8.4 ± 3.2 (8)	86.0 ± 8.0 (7)	13.5 ± 3.9 (5)
	Control (N)	91.0 ± 5.4 (8)	800 ± 100 (8)	9.0 ± 3.0 (8)	76.9 ± 9.0 (7)	39.0 ± 5.5* (5)
C57BL/6	Infected (N)	85.6 ± 6.1 (8)	1 400 ± 100 (6)	7.4 ± 3.7 (9)	35.0 ± 9.8 (6)	19.5 ± 2.7 (5)
	Control (N)	95.9 ± 2.7 (8)	1 500 ± 100 (8)	5.5 ± 3.0 (9)	16.9 ± 8.6* (8)	23.6 ± 12.3 (5)

Data are mean ± S.E.M. m: microsomal fraction; c: cytosolic fraction; (N): number of animals. Values different from those for non-infected controls (Student's *t *test, P < 0.05) are indicated by an asterisk (*). Enzyme activities are expressed as nmol/mg ptn/min (mGST and UGT) and μmol/mg ptn/min (cGST). GSH as nmol/mg ptn; TBARS as nmol MDA/g wet tissue.

#### Effect of *P. berghei *infection on the expression of inducible haem oxygenase (HO-1) in the liver of DBA-2 mice

**Figure 7:**
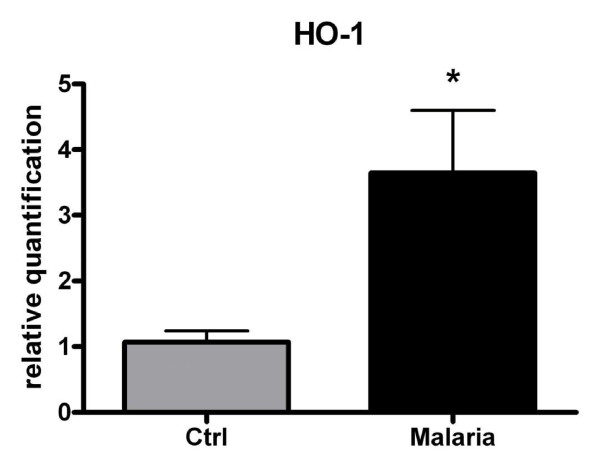
**Effects of *P. berghei *infection on haemoxygenase-1 mRNA expression in the liver of DBA-2 mice**. Expression of HO-1 mRNA in the liver of DBA-2 mice infected with *P.berghei *(ANKA), when parasitaemia rates rose to levels >30%PE on PID20, and respective non-infected controls. Column height represents mean ± S.E.M. for relative levels of mRNA of 6 mice. Differences (Mann-Whitney U test, P < 0.05) between infected (malaria) and non-infected controls (Ctrl) are indicated by an asterisk (*). Quantification of mRNA was by real time RT-PCR.

Disruption of red blood cells during parasite schizogony produces cell-free haemoglobin that is oxidized releasing its haem prosthetic group (free haem), a pro-oxidant and cytotoxic substance. HO-1 plays a major role in the catabolism of free haem and thus it protects host liver cells from free haem deleterious effects during severe malaria infections [36,37]. As shown in Figure 7, levels of HO-1 mRNA in the liver of *P.berghei*-infected DBA-2 mice (parasitaemia >30% PE) were nearly 3-fold levels found in non-infected controls. The concurrent up-regulation of CYP2a5 (Figure 1B) and HO-1 (Figure 7) in the liver of *P.berghei*-infected DBA-2 mice is consistent with the idea advanced by Abu-Bakar *et al *[10] that CYP2a5 takes part in the catabolism of bilirubin, and that coordinated induction of both oxidases results in a balance between overproduction and degradation of bilirubin in response to free haem excess.

#### Effect of *P. berghei *infection on the level of glucose-regulated protein 78 (GRP78) in the mouse liver

**Figure 8:**
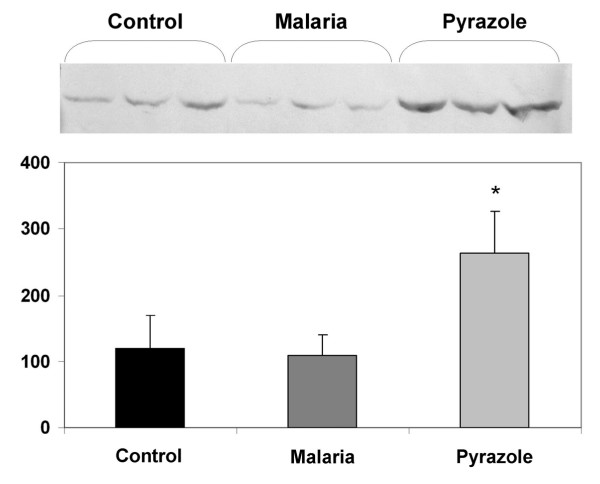
**Effect of *P. berghei *infection on GRP78 protein expression in the liver of DBA-2 mice**. Immunoblot analysis - with a polyclonal antibody against GRP78 - of liver microsomes from female DBA-2 mice infected with *P*. *berghei *(Malaria) on PID20, non-infected controls (Control) and non-infected mice treated with pyrazole (Pyrazole), 100 mg/kg body wt i.p. Densitometric analysis is shown in the lower panel. Height of histogram bars are means ± S.D. of arbitrary optical density units. Difference from non-infected control group is indicated by an asterisk (*, P < 0.05, Mann-Whitney U test). 50 μg of microsomal protein from a different mouse was applied on each lane. The immonoblot shown in the figure is representative of others with similar results.

The overexpression of glucose-regulated protein 78 (GRP78), an endoplasmic reticulum-resident chaperone, has been used as a biomarker of endoplasmic reticulum (ER) stress [9,38]. As shown in Figure 8, densitometric analysis of Western blots revealed that pyrazole (100 mg/kg i.p. 24 h earlier), a hepatotoxic and ER-stress inducing agent, induced GRP78 in the liver of DBA-2 mice. *P.berghei *infection, however, did not elevate the expression of GRP78 protein over the levels found in non-infected controls (Figure 8), thereby indicating that blood stage malaria (parasitaemia >30%PE) did not elicit ER stress in the liver of DBA-2 mice.

## Discussion

Data provided by this study confirmed that *P.berghei *infection induces CYP2a5 (COH) and depresses CYP1a (EROD) and 2b (BROD) activities in the liver of DBA-2 mice [7]. Furthermore, it was also shown that non-lethal (*P.c.chabaudi*) infection up- (CYP2a5) and down- (CYP1a and 2b) modulates CYP activities in the liver of DBA-2 and C57BL/6 mice on the day, and/or shortly before or after the day on which parasitaemia peak was recorded. It is of note that CYP activities remained almost unaltered in C57BL/6 mice infected with *P.berghei*, most of which developed severe neurological symptoms compatible with cerebral malaria. Compared to *P.berghei*-infected DBA-2, however, C57BL/6 mice presented lower parasitaemia rates before dying with cerebral malaria symptoms. Modulation of CYP activities by malaria, therefore, is not necessarily associated with a terminal stage of an invariably lethal infection, because, on one side, it was not observed in mice (C57BL/6) developing a severe and deadly cerebral malaria, and, on the other side, it occurred near the parasitaemia peak in animals with non-lethal infections. Taken together, findings presented here suggested that high parasitaemia rate was a major clinical feature associated with infection-produced alterations of liver CYP activities regardless of whether infection further progressed toward the death (*P.berghei*) or cure (*P.chabaudi*) of malarious mice. Additionally, necropsy data showed that livers of *P.chabaudi*-infected mice, due to a continuous accumulation of malaria pigment (haemozoin), became even darker and more enlarged after the day of parasitaemia peak, when CYP activities had returned to the levels recorded in non-infected controls. The foregoing observation suggested that up- (CYP2a5) and down- (CYP1a and 2b) regulations of liver CYP activities during malaria infections do not seem to be associated with accumulation of haemozoin in the hepatic tissue.

CYP2a5 is induced by phenobarbital, pyrazole, virus and bacterial hepatitis as well as by liver fluke (*Fasciola hepatica *and *Opisthorchis viverrini*) infections [39-41]. Recently, it was described that CYP2a5 is induced in DBA-2 and C57BL/6 mice infected with *P*. *berghei *ANKA[7]. All aforementioned infections and the prototypical CYP2a5-inducer pyrazole - but not phenobarbital - produced extensive liver injury and a marked rise in serum levels of ALT and AST as well [9]. The expression of CYP2a5 in a glucose-6-phosphate dehydrogenase (G6PD)-deficient mouse model of oxidative stress was studied by Nichols and Kirby [42]. The G6PD-deficient mouse exhibits increased susceptibility to oxidative stress and the authors found that treatment of wild type and G6PD-deficient mice with pyrazole for 72 hours led to a greater degree of induction of CYP2a5 in the deficient animals. TBARS and other markers of oxidative stress, however, were not altered by pyrazole either in wild or in G6PD-deficient mice. On the other side, oxidative stress markers were enhanced, whereas CYP2a5 was not induced by treatment of wild type or G6PD-deficient mice with menadione, a pro-oxidant agent. Nichols and Kirby [42] also noted that pyrazole produced a greater increase in GRP78 protein in G6PD-deficient mice than in wild type mice, and that menadione did not alter GRP78 levels either in wild type or in G6PD-deficient animals. Based on these findings, the authors suggested that generalized cellular oxidative stress does not play a key role in the up-regulation of CYP2a5 and that other factors related to G6PD-deficiency such as ER stress may be involved.

Results from the present study indicated that levels of the putative oxidative stress marker TBARS were increased, while expression of GRP78 remained unchanged in the liver of *P.berghei*-infected DBA-2 mice. The effects of malaria on TBARS and GRP78 expression were similar to the effects of menadione, a pro-oxidant agent non-inducer of CYP2a5, and contrasted with the effects of pyrazole, a prototypical inducer of CYP2a5. Therefore, it seems difficult reconciling all aforementioned studies findings through a common hypothesis on the role of oxidative stress and ER stress on CYP2a5 induction by chemicals and infections. Nonetheless, it should be borne in mind that lack of GRP78 induction does not necessarily imply that malaria elicited no ER stress. Although being considered a marker of ER stress, GRP78 over-expression is in fact a homeostatic response that protects the cell against damage caused by ER dysfunction or stress. If GRP78 were not up-regulated, ER stress would result in more marked ER accumulation of unfolded proteins and more severe hepatocellular injury. This interpretation is consistent with elevated serum ALT and AST levels noted in *P.berghei*-infected DBA-2 mice [7], an indication that enhanced oxidative stress during malaria may have caused liver cell injury.

HO-1 is an inducible and rate-limiting enzyme of haem catabolic pathway. It catalyzes the opening of porphyrin ring to form biliverdin that is subsequently converted to bilirubin by biliverdin reductase. Circulating free haem is a potent oxidant and cytotoxic agent and thus up-regulation of HO-1 expression by oxidative stress, heavy metals and a variety of other inducers is regarded as a protective response. Although being a protective antioxidant compound, bilirubin is very hydrophobic and may also cause toxicity when it is accumulated in tissues due to impaired conjugation and excretion (e.g. neurotoxicity in neonatal jaundice and cholestatic diseases). The main route of bilirubin elimination involves glucuronidation mediated by UGT1A1 and excretion of conjugated metabolites into the bile. Nonetheless, it has been suggested that oxidative metabolism of bilirubin would be an important alternative route of elimination when glucuronidation is impaired [10]. Based on the observation that CYP2a5 is induced by substances known to affect haem balance, including metals and porphyrinogenic agents [43], Abu Bakar *et al *[10] suggested that CYP2a5 plays a role in the oxidative metabolism of bilirubin. Moreover, the authors pointed out that the foregoing CYP2a5 inducers also up-regulated HO-1, and that CYP2a5 over-expression is often associated with a decrease of total CYP content, which in turn has been shown to be associated with HO-1 induction as well. A hypothesis was advanced that a coordinated regulation of CYP2a5 and HO-1 expressions contributes to achieving a balance between bilirubin production and elimination. The concurrent induction of CYP2a5 and HO-1 in the liver of *P*. *berghei*-infected DBA-2 mice is consistent with this view [10].

Contrasting with the up- and down-modulation of CYPs, activities of liver conjugation enzymes (UGT and microsomal and cytosolic GST) remained unchanged in *P.berghei*-infected mice. Carvalho *et al *[6] also found that UGT activity was unaltered in liver microsomes of Swiss Webster mice infected with *P*. *berghei*. Murdoch *et al *[44], however, evaluated phenol conjugation in perfused rat livers and noted that *P.berghei *infection decreased phenol glucuronide formation. Along the same line, Ismail *et al *[45] described that malaria impaired glucuronidation of paracetamol in rats in vivo. In both cases, ex vivo and in vivo reductions in phenol and paracetamol glucuronidations may have been due to a lower availability of the co-substrate uridine diphosphoglucuronic acid (UDPGA) in the liver of infected rats. As pointed out by Murdoch *et al *[44], UDPGA availability depends on hepatic glycogen stores, which were partially depleted in malarious rats. Therefore, even if expression and catalytic activity of UGT remains unaltered, a lower availability of the co-substrate UDPGA in the liver of malarious individuals may eventually reduce the rate of glucuronidation reactions.

There are few data on the effects of rodent malaria on the expression and activity of GSTs in the host liver. Results from this study with *P.berghei *ANKA infections are at variance with those reported by Ahmad and Srivastava [46] for mice infected with *Plasmodium yoelii nigeriensis*. The authors found a decline of microsomal and cytosolic GST activities in the liver and spleen of infected mice that were to some extent reversed by treatment with mefloquine and menadione. The differences between results from this study and those reported by Ahmad and Srivastava [46] are possibly explained by differences regarding parasite species, murine strains and severities of malaria infection.

During malaria infection both hosts and parasites are under oxidative stress and reactive oxygen species (ROS, e.g., superoxide anions and hydroxyl radicals) are produced by host's activated neutrophils and by the degradation of haemoglobin by parasites as well. Although killing the parasite, enhanced ROS production also contributes to pathological changes in the host including red blood cell sequestration, cerebral pathology, anemia, and respiratory distress. Data from this study showed that levels of TBARS - a marker for lipid peroxidation and oxidative stress - were enhanced in the liver of DBA-2 mice infected with *P.berghei*, thereby supporting the view that hepatic tissue is under oxidative stress during severe malaria infection. No alteration of TBARS levels was found in the liver of infected C57BL/6 mice. On the day of sacrifice, however, infected C57BL/6 mice exhibited lower parasitaemia rates and less darkened and enlarged livers than infected DBA-2 mice. Levels of GSH in the liver, on the other side, were reduced by infection in C57BL/6 but not in DBA-2. It is of note that background levels (i.e., non-infected control levels) of GSH in the DBA-2 strain were higher than in the C57BL/6 strain. It is not clear why hepatic GSH levels were depleted by malaria in C57BL/6 mice but not in DBA-2 mice. A number of factors can affect the availability of GSH (a powerful antioxidant) in the liver and owing to this fact a decline of the ratio between reduced (GSH) and oxidized (GSSG) glutathione is a more reliable indicator of imbalance between pro-oxidant and antioxidant mechanisms (oxidative stress) than a decrease of GSH levels.

Nonetheless, data from this study strongly suggest that, owing to their lower levels of GSH, infected C57BL/6 mice are more vulnerable to hepatic injury (including DNA damage) caused by electrophiles than non-infected mice.

It is well known that NO production in response to secretion of pro-inflammatory cytokines during infection and inflammation is mediated by an up-regulation of iNOS [47]. In human malaria, NO has been suggested to have a dual role. In *P*. *falciparum *malaria, despite mediating the intra-hepatic killing of the parasite, NO is thought to contribute to the pathogenesis of the disease [47]. In rodent malaria, however, the role of NO is still unclear. Despite the general idea that it depends on the parasite-host relationship, iNOS mRNA induction in rodent malaria may vary according to the stage of infection, the degree of parasitaemia, the species and strain of *Plasmodium *and the target organ or tissue examined [48]. In this study, no enhancement of serum NO was found in *P.berghei*-infected mice while slight increases of serum levels of NO were observed near the day of parasitaemia peak in *P.chabaudi*-infected animals. Nonetheless, levels of iNOS mRNA in the liver of *P.berghei*-infected mice were higher than levels in non-infected controls, thereby indicating that iNOS expression was up-regulated by lethal malaria. A possible explanation for unchanged serum levels of NO, in the presence of over-expression of iNOS in hepatic and splenic tissues, is an increase in NO scavenging by free haemoglobin in the blood of infected mice. Nitric oxide binds avidly to haem iron, and haemoglobin (Hb) is released into the circulation when red blood cells (RBC) are disrupted during parasite schizogony. Along this line, Sobolewski *et al *[49] found that plasma levels of free Hb were rather high in *P.berghei*-infected mice (e.g. on PID6), and additionally demonstrated in vitro that cell free Hb is at least 1 000 times more efficient in scavenging NO than the intracellular Hb. It should be pointed out that intravenous infusions of cell free haemoglobin and chronic haemolysis due to sickle-cell disease limit NO bioavailability as well [50]. The induction of iNOS mRNA expression in *P.berghei*-infected mice strongly suggested that malaria infection elicited NO production in the hepatic tissue. The idea that NO is a mediator of the down-modulation of CYP expression by inflammatory stimuli is plausible because NO binds haem proteins and inhibits catalytic activities of CYPs in in vitro test systems [16,17]. Nonetheless, Khatsenko and coworkers' hypothesis has not been supported by data provided by other authors. Sewer *et al *[18], for instance, reported that LPS depressed the expression of CYP2c29, 2e1 and 3a11 in iNOS knockout mice. More recently, however, Morgan and coworkers [2,51] showed that NO over-production caused a selective depression of CYP2b rather than an unspecific down-regulation of CYPs. Furthermore, Lee *et al *[51] showed that CYP2b underwent a post-transcriptional NO-dependent ubiquitination and proteosomal degradation and suggested that these events are triggered by a modification of aminoacid residues of CYP2b proteins by reactive nitrogen species. If this view holds true, it seems fair to think that NO overproduction arising from iNOS up-regulation in the hepatic tissue is the mechanism, or one of the mechanisms, by which malaria depressed CYP2b activity in the mouse liver.

The mechanism by which malaria down-modulates the expression and activity of other CYP isoforms is still unclear. Gu *et al *[52] suggested that NFκB activation could play a role in the down-modulation of CYPs by inflammatory and infectious diseases and malaria was reported to enhance TLR-mediated pro-inflammatory responses [53]. Therefore, the idea that NFκB activation mediates the down-modulation of liver CYPs by blood stage malaria seems plausible.

Finally, it should be borne in mind that there are differences in the pathology produced by *P*. *berghei *and *P.chabaudi *in murine hosts and that caused by *P*. *falciparum *and *Plasmodium vivax *in humans. Results from this study showed that up- and down-regulation of CYP activities during *P.berghei *and *P.chabaudi *infections occurred when parasitaemia rates were high. Compared to the hyperparasitaemia noted in murine infections, parasitaemia rates in human *P.falciparum *infection are low due to the adherence of trophozoite/schizont-infected erythrocytes to the endothelium of capillaries and venules. Nonetheless, it was reported that, in patients with *P.falciparum *malaria, clearance rates of quinine [54] and caffeine [55] were slower thereby suggesting that drug metabolism is depressed during human malaria as well. There are only a few studies on the influence of malaria on drug metabolism in humans and more data are needed on the alterations of drug kinetics in *P.falciparum *and also in *P.vivax *malaria. At any rate, data presented here demonstrated that liver CYP activities are modulated during murine lethal and non-lethal infections and it seems fair to think that this modulation of enzymes involved in drug metabolism also occurs in human falciparum malaria.

## Conclusion

In conclusion, results from this study showed that down-regulation of CYP1a and 2b, as well as up-regulation of CYP2a5 occur in both lethal and non-lethal murine malaria. It was also demonstrated that alterations of CYP activities in murine malaria occurred only when parasitaemia rates were high, and apparently were not associated with liver enlargement and haemozoin accumulation. Since iNOS was induced in the liver of *P.berghei*-infected mice, a contribution of NO for depression of CYP2b activity cannot be ruled out. No induction of GRP78 was found in *P.berghei*-infected mice, a finding that suggests that CYP2a5 up-modulation may occur in the absence of ER stress. Finally, results from this study are consistent with the view that HO-1 and CYP2a5 are concurrently up-regulated in the mouse liver.

## Competing interests

The authors declare that they have no competing interests.

## Authors' contributions

ACAXD-O conceived the study and participated in its design, statistical analysis and results interpretation, supervised the performance of experiments at the bench and drafted the manuscript; RSC carried out real-time RT-PCR assays; FHP carried out parasite infections, immunoblotting and glutathione measurement; HST, LSG and CMS prepared liver subcellular fractions, and performed enzyme assays and TBARs assay; FJRP contributed in the design of the study and in the analysis and interpretation of data. All authors read and approved the final manuscript.

## References

[B1] Morgan ET Regulation of cytochrome P450 by inflammatory mediators: why and how? Drug Metab Dispos 2001 29 207 212 11181485

[B2] Aitken AE Richardson TA Morgan ET Regulation of drug-metabolizing enzymes and transporters in inflammation Annu Rev Pharmacol Toxicol 2006 46 123 149 10.1146/annurev.pharmtox.46.120604.141059 16402901

[B3] Alvares AP Ueng TH Scheibel LW Hollingdale MR Impairment of hepatic cytochrome P-450 dependent monooxygenases by the malaria parasite *Plasmodium berghei* Mol Biochem Parasitol 1984 13 277 282 10.1016/0166-6851(84)90119-1 6396516

[B4] Uhl K Grace JM Kocisko DA Jennings BT Michell AL Brewer TG Effects of *Plasmodium berghei *infection on cytochromes P-450 2E1 and 3A2 Eur J Drug Metab Pharmacokinet 1999 24 169 176 10510746 10.1007/BF03190365

[B5] Poça KS De-Oliveira AC Santos MJ Paumgartten FJ Malaria infection modulates effects of genotoxic chemicals in the mouse bone-marrow micronucleus test Mutat Res 2008 649 28 33 17851116 10.1016/j.mrgentox.2007.07.006

[B6] Carvalho RS Friedrich K De-Oliveira AC Suarez-Kurtz G Paumgartten FJ Malaria downmodulates mRNA expression and catalytic activities of CYP1A2, 2E1 and 3A11 in mouse liver Eur J Pharmacol 2009 616 265 269 10.1016/j.ejphar.2009.05.030 19501084

[B7] De-Oliveira ACAX Da-Matta AC Paumgartten FJR *Plasmodium berghei *(ANKA): Infection induces CYP2A5 and 2E1 while depressing other CYP isoforms in the mouse liver Exp Parasitol 2006 113 256 261 10.1016/j.exppara.2006.01.013 16540109

[B8] Gilmore WJ Hartmann G Piquette-Miller M Marriott J Kirby GM Effects of lipopolysaccharide-stimulated inflammation and pyrazole-mediated hepatocellular injury on mouse hepatic Cyp2a5 expression Toxicology 2003 184 211 216 10.1016/S0300-483X(02)00581-4 12499123

[B9] Gilmore WJ Kirby GM Endoplasmic reticulum stress due to altered cellular redox status positively regulates murine hepatic CYP2A5 expression J Pharmacol Exp Ther 2004 308 600 608 10.1124/jpet.103.060111 14610226

[B10] Abu-Bakar A Moore MR Lang MA Evidence for induced microsomal bilirubin degradation by cytochrome P450 2A5 Biochem Pharmacol 2005 70 1527 1535 10.1016/j.bcp.2005.08.009 16183037

[B11] Francis SE Sullivan DJ Jr Goldberg DE Hemoglobin metabolism in the malaria parasite *Plasmodium falciparum* Annu Rev Microbiol 1997 51 97 123 10.1146/annurev.micro.51.1.97 9343345

[B12] Omodeo-Salè F Motti A Dondorp A White NJ Taramelli D Destabilization and subsequent lysis of human erythrocytes induced by *Plasmodium falciparum *haem products Eur J Haematol 2005 74 324 332 10.1111/j.1600-0609.2004.00352.x 15777345

[B13] Brinkmann V Kaufmann SHE Simon MM Fischer H Role of macrophages in malaria: O2 metabolite production and phagocytosis by splenic macrophages during lethal *Plasmodium berghei *and self-limiting *Plasmodium yoelii *infection in mice Infect Immun 1984 44 743 746 6373617 10.1128/iai.44.3.743-746.1984 PMC263687

[B14] Maines MD Trakshel GM Kutty RK Characterization of two constitutive forms of rat liver microsomal heme oxygenase. Only one molecular species of the enzyme is inducible J Biol Chem 1986 261 411 419 3079757

[B15] Tenhunen R Marver HS Schmid R The enzymatic conversion of heme to bilirubin by microsomal heme oxygenase Proc Natl Acad Sci USA 1968 61 748 755 10.1073/pnas.61.2.748 4386763 PMC225223

[B16] Khatsenko OG Gross SS Rifkind AB Vane JR Nitric oxide is a mediator of the decrease in cytochrome P450-dependent metabolism caused by immunostimulants Proc Natl Acad Sci USA 1993 90 11147 11151 10.1073/pnas.90.23.11147 7504296 PMC47939

[B17] Khatsenko O Kikkawa Y Nitric Oxide Differentially Affects Constitutive Cytochrome P450 Isoforms in Rat Liver J Pharmacol Exp Ther 1997 250 1463 1470 9067336

[B18] Sewer MB Barclay TB Morgan ET Down-regulation of cytochrome P450 mRNAs and proteins in mice lacking a functional NOS2 gene Mol Pharmacol 1998 54 273 279 9687568 10.1124/mol.54.2.273

[B19] Li YQ Prentice DA Howard ML Mashford ML Desmond PL Bilirubin and bile acids may modulate their own metabolism via regulating uridine diphosphate-glucuronosyltransferase expression in the rat J Gastroenterol Hepatol 2000 15 865 867 10.1046/j.1440-1746.2000.02223.x 11022826

[B20] De-Oliveira ACAX Ribeiro-Pinto LF Paumgartten FJR In vitro inhibition of CYP2B1 monooxygenase by myrcene and other monoterpenoid compounds Toxicol Lett 1997 92 39 46 10.1016/S0378-4274(97)00034-9 9242356

[B21] Fowler BA Kleinow KM Squibb KS Lucier GW Hayes AW Wallace Hayes A Organelles as Tools in Toxicology Principles and Methods of Toxicology 1994 Chapter 33 3 222 223

[B22] Abel EL Bammler TK Eaton DL Biotransformation of methyl parathion by glutathione S-transferase Toxicol Sci 2004 79 224 232 10.1093/toxsci/kfh118 15103050

[B23] Bradford MM A rapid and sensitive method for the quantitation of microgram quantities of protein utilizing the principle of protein dye binding Anal Biochem 1976 72 248 254 10.1016/0003-2697(76)90527-3 942051

[B24] Kennedy SW Jones SP Simultaneous measurement of cytochrome P450 catalytic activity and total protein concentration with fluorescent plate reader Anal Biochem 1994 222 217 223 10.1006/abio.1994.1476 7856852

[B25] van Iersel M Walters DG Price RJ Lovell DP Lake BG Sex and strain differences in mouse hepatic microsomal coumarin-7-hydroxylase activity Food Chem Toxicol 1994 32 387 390 10.1016/0278-6915(94)90078-7 8206435

[B26] Bock KW Burchell B Dutton GJ Hanninen O Mulder GJ Owens IS Siest G Tephly TR UDP-glucuronosyltransferase activities Guidelines for consistent interim terminology and assay conditions Biochem Pharmacol 1983 32 953 955 10.1016/0006-2952(83)90610-X 6404284

[B27] Martin ST Black SD Detergent effects in rabbit liver microsomal UDP-glucuronosyltransferase studied by means of a continuous spectrophotometric assay with *p*-nitrophenol Biochem Biophys Res Commun 1994 200 1093 1098 10.1006/bbrc.1994.1562 8179587

[B28] Habig WH Pabst MJ Jakoby WB Glutathione S-transferases The first enzymatic step in mercapturic acid formation J Biol Chem 1974 249 7130 7139 4436300

[B29] Cao Z Li Y The chemical inducibility of mouse cardiac antioxidants and phase 2 enzymes in vivo Biochem Biophys Res Commun 2004 17 1080 1088 10.1016/j.bbrc.2004.03.156 15094379

[B30] Zhu H Li Y Trush MA Characterization of benzo[a]pyrene quinone-induced toxicity to primary cultured bone marrow stromal cells from DBA-2 mice: potential role of mitochondrial dysfunction Toxicol Appl Pharmacol 1995 130 108 120 10.1006/taap.1995.1015 7530864

[B31] Cao Z Hardej D Trombetta LD Trush MA Li Y Induction of cellular glutathione and glutathione S-transferase by 3H-1,2-dithiole-3-thione in rat aortic smooth muscle A10 cells: protection against acrolein-induced toxicity Atherosclerosis 2003 166 291 302 10.1016/S0021-9150(02)00331-3 12535742

[B32] Hermes-Lima M Wang EM Schulman HM Storey KB Ponka O Deoxyribose degradation catalyzed by Fe(III)-EDTA. Kinetic aspects and potential uselfulness for sub-micromolar iron measurements Mol Cell Biochem 1994 137 65 73 10.1007/BF00926041 7845380

[B33] Schmidt HH Wilke P Evers B Böhme E Enzymatic formation of nitrogen oxides from L-arginine in bovine brain cytosol Biochem Biophys Res Commun 1989 165 284 291 10.1016/0006-291X(89)91067-X 2590227

[B34] Laemmli UK Cleavage of structural proteins during the assembly of the head of bacteriophage T4 Nature 1970 227 680 685 10.1038/227680a0 5432063

[B35] de Souza JB Riley EM Cerebral malaria: the contribution of studies in animal models to our understanding of immunopathogenesis Microbes Infect 2002 4 291 300 10.1016/S1286-4579(02)01541-1 11909739

[B36] Ferreira A Balla J Jeney V Balla G Soares MP A central role for free heme in the pathogenesis of severe malaria: the missing link J Mol Med 2008 86 1097 1111 10.1007/s00109-008-0368-5 18641963

[B37] Seixas E Gozzelino R Chora A Ferreira A Silva G Larsen R Rebelo S Penido C Smith NR Coutinho A Soares MP Heme oxygenase-1 affords protection against noncerebral forms of severe malaria Proc Natl Acad Sci USA 2009 106 15837 15842 10.1073/pnas.0903419106 19706490 PMC2728109

[B38] Hosoi T Saito A Kume A Okuma Y Nomura Y Ozawa K Vanadate inhibits endoplasmic reticulum stress responses Eur J Pharmacol 2008 594 44 48 10.1016/j.ejphar.2008.07.034 18700142

[B39] Kirby GM Pelkonen P Vatanasapt V Camus A-M Wild CP Lang MA Association of liver fluke (*Opistorchis viverrini*) infestation with increased expression of cytochrome P450 and carcinogen metabolism in male hamster liver Mol Carcinog 1994 11 81 89 10.1002/mc.2940110205 7916996

[B40] Montero R Serrano L D'avila VM Ito A Plancarte A Infection of rats with *Taenia taeniformis *metacestodes increases hepatic CYP450, induces the activity of CYP1A1, CYP2B1 and COH isoforms and increases the genotoxicity of the procarcinogens benzo[a]pyrene, cyclophosphamide and aflatoxin B(1) Mutagenesis 2003 18 211 216 10.1093/mutage/18.2.211 12621079

[B41] Richardson TA Sherman M Antonovic L Kardar SS Strobel HW Kalman D Morgan ET Hepatic and renal cytochrome p450 gene regulation during *citrobacter rodentium *infection in wild-type and toll-like receptor 4 mutant mice Drug Metab Dispos 2006 34 354 356 16339354 10.1124/dmd.105.007393 PMC1382008

[B42] Nichols KD Kirby GM Expression of cytochrome P4502A5 in a glucose-6-phosphate dehydrogenase-deficient mouse model of oxidative stress Biochem Pharmacol 2008 75 1230 1239 10.1016/j.bcp.2007.10.032 18068688

[B43] Salonpaa P Krause K Pelkonen O Raunio H Up-regulation of CYP2A5 expression by porphyrinogenic agents in mouse liver Naunyn Schmiedebergs Arch Pharmacol 1995 351 446 452 10.1007/BF00169087 7543189

[B44] Murdoch RT Ghabrial H Smallwood RA Morgan DJ Effects of malaria on phenol conjugation pathways in perfused rat liver Biochem Pharmacol 1992 43 1229 1234 10.1016/0006-2952(92)90496-6 1562275

[B45] Ismail S Kokwaro GO Back DJ Edwards G Effect of malaria infection on the pharmacokinetics of paracetamol in rat Xenobiotica 1994 24 527 533 10.3109/00498259409043255 7975718

[B46] Ahmad R Srivastava AK Effect of *Plasmodium yoelii nigeriensis *infection on hepatic and splenic glutathione-S-transferase(s) in Swiss albino and db/+ mice: efficacy of mefloquine and menadione in antimalarial chemotherapy Parasitology 2007 134 931 938 10.1017/S003118200700234X 17352848

[B47] Brunet LR Nitric oxide in parasitic infections Int Immunopharmacol 2001 1 1457 1467 10.1016/S1567-5769(01)00090-X 11515811

[B48] Nahrevanian H Immune effector mechanisms of the nitric oxide pathway in malaria: cytotoxicity versus cytoprotection Braz J Infect Dis 2006 10 283 292 10.1590/S1413-86702006000400014 17293913

[B49] Sobolewski P Gramaglia I Frangos JA Intaglietta M Heyde H van der *Plasmodium berghei *resists killing by reactive oxygen species Infect Immun 2005 3 6704 6710 10.1128/IAI.73.10.6704-6710.2005 PMC1230976 16177347

[B50] Reiter CD Wang X Tanus-Santos JE Hogg N Cannon RO Schechter AN Gladwin MT Cell-free hemoglobin limits nitric oxide bioavailability in sickle-cell disease Nat Med 2002 8 1383 1389 10.1038/nm799 12426562

[B51] Lee CM Kim BY Li L Morgan ET Nitric oxide-dependent proteasomal degradation of cytochrome P450 2B proteins J Biol Chem 2008 283 889 898 10.1074/jbc.M708821200 17993647 PMC2185771

[B52] Gu X Ke S Liu D Sheng T Thomas PE Rabson AB Gallo MA Xie W Tian Y Role of NF-kappaB in regulation of PXR-mediated gene expression: a mechanism for the suppression of cytochrome P-450 3A4 by proinflammatory agents J Biol Chem 2006 281 17882 17889 10.1074/jbc.M601302200 16608838

[B53] Franklin BS Parroche P Ataíde MA Lauw F Ropert C de Oliveira RB Pereira D Tada MS Nogueira P da Silva LH Bjorkbacka H Golenbock DT Gazzinelli RT Malaria primes the innate immune response due to interferon-gamma induced enhancement of toll-like receptor expression and function Proc Natl Acad Sci USA 2009 106 5789 5794 10.1073/pnas.0809742106 19297619 PMC2657593

[B54] Trenholme GM Williams RL Rieckman KH Frischer H Carson PE Quinine disposition during malaria and during induced fever Clin Pharmacol Ther 1976 19 459 467 773582 10.1002/cpt1976194459

[B55] Akinyinka OO Sowunmi A Honeywell R Renwick AG The effects of acute falciparum malaria on the disposition of caffeine and the comparison of saliva and plasma-derived pharmacokinetic parameters in adult Nigerians Eur J Clin Pharmacol 2000 56 159 165 10.1007/s002280050735 10877011

